# Supplementary material for: A machine learning approach using partitioning around medoids clustering and random forest classification to model groups of farms in regard to production parameters and bulk tank milk antibody status of two major internal parasites in dairy cows
Source: PLoS One. 2022 Jul 11;17(7):e0271413. doi: 10.1371/journal.pone.0271413 (PMC9273072; doi:10.1371/journal.pone.0271413)
Supplement: S1 Table — A complete list of implemented R packages in alphabetical order. (PDF) [file pone.0271413.s001.pdf]

# List of R packages, alphabetical order

| Data editing |
|---|
| bayesboot [1] |
| dplyr [2] |
| ggpubr [3] |
| lubridate [4] |
| plyr [5] |
| tidyverse [6] |
| **Analyses** |
| cluster [7] |
| ggplot2 [8] |
| randomForest [9] |
| rfPermute [10] |
| ROCR [11] |
| Rtsne [12-14] |
| vip [15] |

References


1. Baath R. bayesboot: an implementation of Rubin's (1981) bayesian bootstrap. R package version 0.2.2. 2018. https://CRAN.R-project.org/package=bayesboot
2. Wickham H, Francois R, Henry L, Müller K. dplyr: A grammar of data manipulation. R package version 1.0.7. 2021. https://CRAN.R-project.org/package=dplyr
3. Kassambara A. ggpubr: ggplot2' based publication ready plots. R package version 0.4.0. https://CRAN.R-project.org/package=ggpubr. 2020.
4. Grolemund G, Wickham H. Dates and times made easy with lubridate. J Stat Softw. 2011;40:1-25.
5. H W. The split-apply-combine strategy for data analysis. J Stat Softw. 2011;40:1-29. http://www.jstatsoft.org/v40/i01/
6. Wickham H, Averick M, Bryan J, Chang W, D'Agostino McGowan L, Francois R, Grolemund G, Hayes A, Henry L, Hester J, Kuhn M, Pedersen TL, Miller E, Milton Bache S, Müller K, Ooms J, Robinson D, Seidel DP, Spinu V, Takahashi K, Vaughan D, Wilke C, Woo K, H Y. Welcome to the tidyverse. J Open Source Softw. 2019;4:1686.
7. Maechler M, Rousseeuw PJ, Struyf A, Hubert M, K H. cluster: Cluster analysis basics and extensions. R package version 2.1.0; 2021. https://CRAN.R-project.org/package=cluster
8. Wickham H. ggplot2: elegant graphics for data analysis: Springer-Verlag, New York; 2016. https://ggplot2.tidyverse.org
9. Liaw A, Wiener M. Classification and regression by randomForest. R News. 2002;2:18-22.
10. Archer E. rfPermute: Estimate permutation p-values for random forest importance Metrics. R package version 2.5.1. 2022. https://CRAN.R-project.org/package=rfPermute
11. Sing T, Sander O, N B, T L. ROCR: visualising classifier performance in R. Bioinformatics. 2005;21:7881. http://rocr.bioinf.mpi-sb.mpg.de
12. Krijthe j. Rtsne: t-distributed stochastic neighbor embedding using a Barnes-Hut implementation. 2015. https://github.com/jkrijthe/Rtsne
13. van der Maaten L, Hinton G. Visualizating high-dimensional data using t-SNE. J Mach Learn Res. 2008;9:2579-605.

14. van der Maaten L. Accelerating t-SNE using tree-based algorithms. J Mach Learn Res. 2014;15:3221-45.
15. Greenwell B, Boehmke b. Variable importance plots-an introduction to the vip package. The R Journal. 2020;12:343-66.
