# Supplementary figures and images for: A machine learning approach using partitioning around medoids clustering and random forest classification to model groups of farms in regard to production parameters and bulk tank milk antibody status of two major internal parasites in dairy cows
Source: PLoS One. 2022 Jul 11;17(7):e0271413. doi: 10.1371/journal.pone.0271413 (PMC9273072; doi:10.1371/journal.pone.0271413)

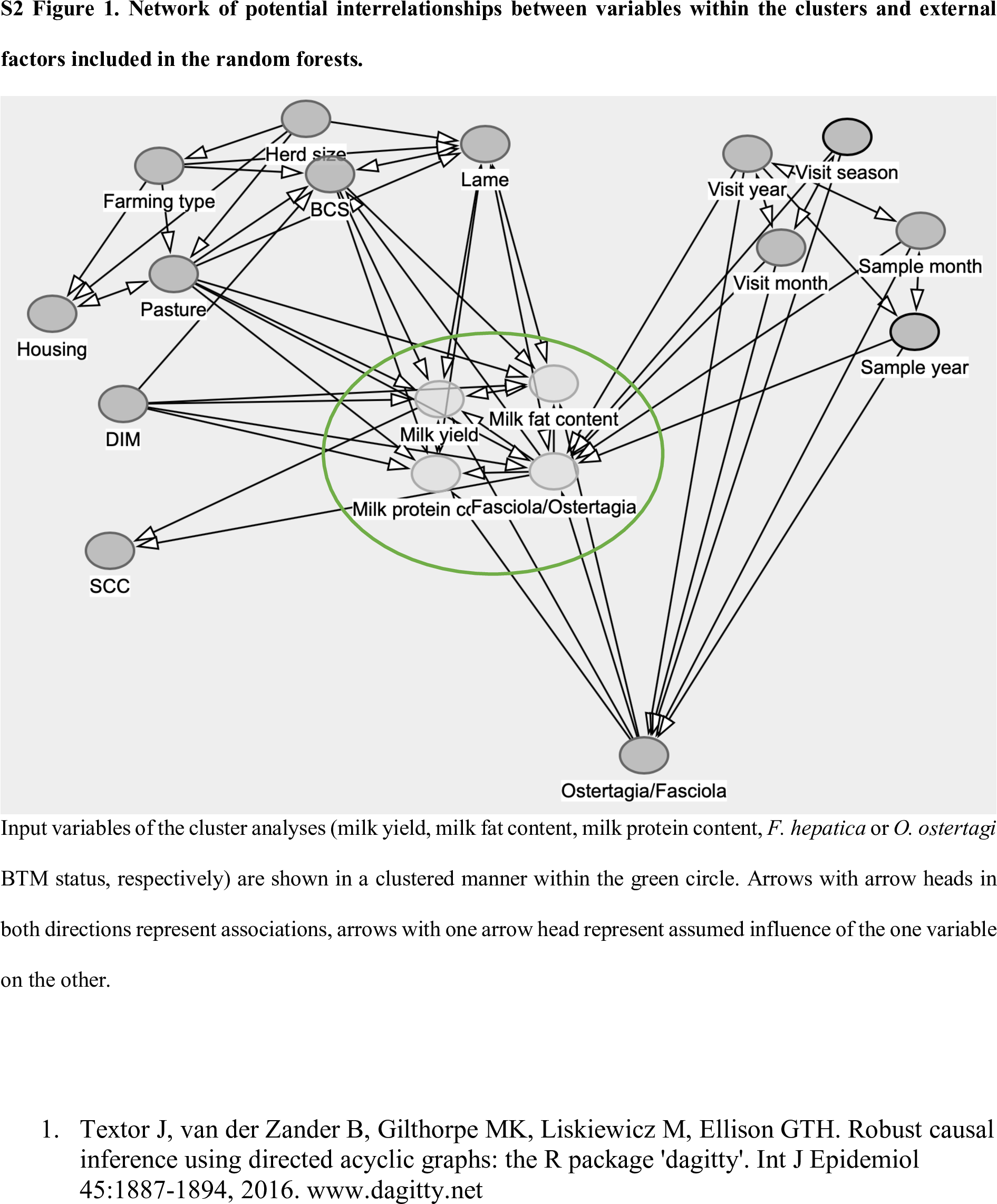

Supplement: S1 Fig — Input variables of cluster analyses and potential associations with external factors. (TIF) [file pone.0271413.s002.tif]

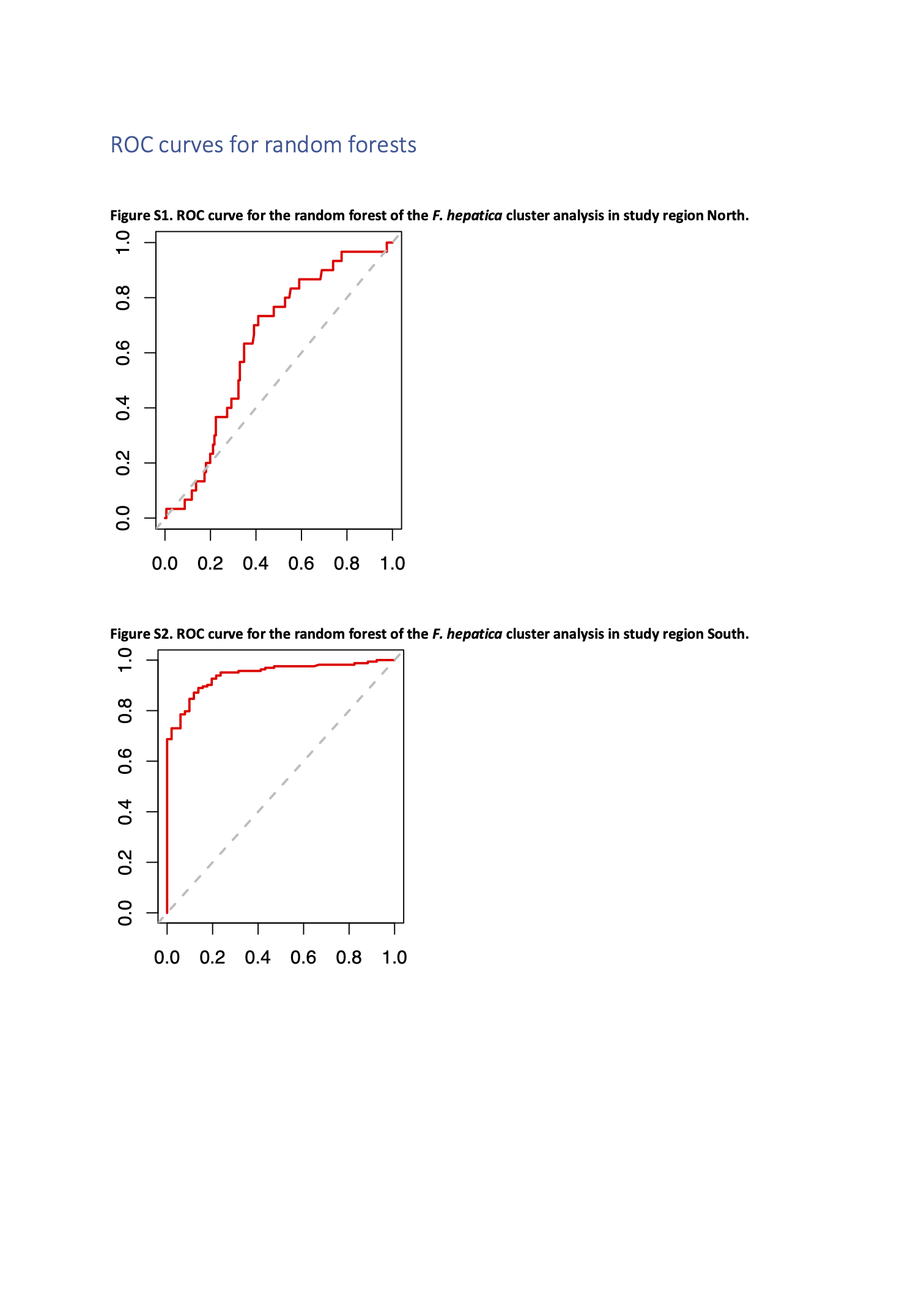

Supplement: S2 Fig — (TIF) [file pone.0271413.s003.tif]

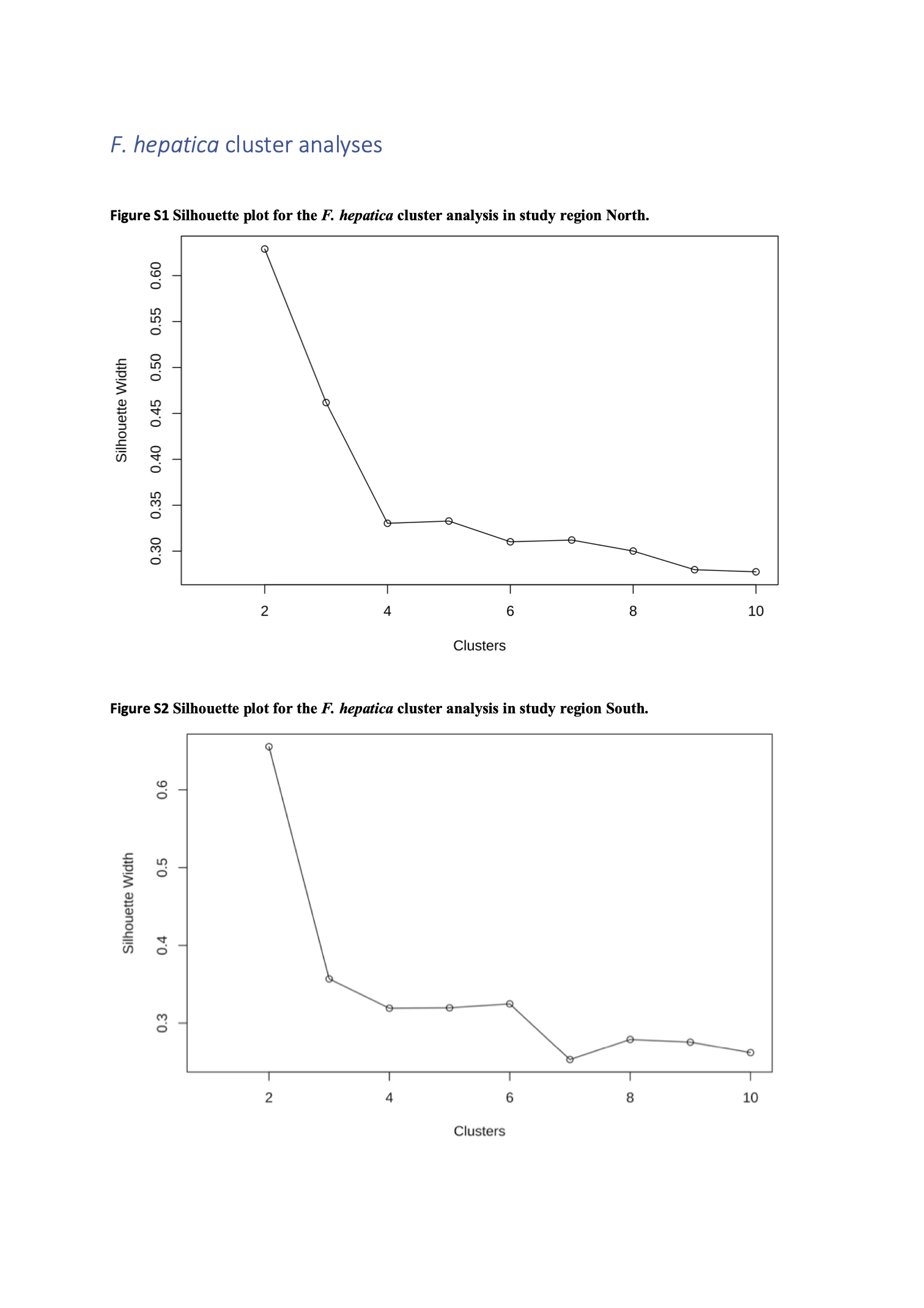

Supplement: S3 Fig — Across all cluster analyses, silhouette plots suggested k = 2 clusters to be the most appropriate number of clusters given the underlying data. (TIF) [file pone.0271413.s004.tif]
