# Supplementary material for: A machine learning approach using partitioning around medoids clustering and random forest classification to model groups of farms in regard to production parameters and bulk tank milk antibody status of two major internal parasites in dairy cows
Source: PLoS One. 2022 Jul 11;17(7):e0271413. doi: 10.1371/journal.pone.0271413 (PMC9273072; doi:10.1371/journal.pone.0271413)
Supplement: Attachment — Submitted filename: Response to Reviewers.docx [file pone.0271413.s012.docx]

Dear Editor

Dear Reviewer 1

Dear Reviewer 2

*We wish to express our gratitude to each of you for taking the time and effort to critically revise our manuscript. We appreciate the overall supportive feedback and feel that the raised points are worth being addressed. Please find below your remarks and our replies. While working on the improvement of this work, we have applied great care to comply with your comments. If there are any further points that need to be modified, we are happy to discuss.*

1. Please ensure that your manuscript meets PLOS ONE's style requirements, including those for file naming. The PLOS ONE style templates can be found at

https://journals.plos.org/plosone/s/file?id=wjVg/PLOSOne_formatting_sample_main_body.pdf and

<https://journals.plos.org/plosone/s/file?id=ba62/PLOSOne_formatting_sample_title_authors_affiliations.pdf>

*We have modified the naming for the supplementary files both within the main body of the manuscript as well as of the uploaded files. We furthermore have effected some minor modifications within the references section.*

2. Thank you for stating the following financial disclosure:

“Farm visits and data collection in the context of the underlying cross-sectional study were financially supported by the German Federal Ministry of Food and Agriculture (BMEL) through the Federal Office for Agriculture and Food (BLE) grant number 2814HS008.”

Please state what role the funders took in the study. If the funders had no role, please state: "The funders had no role in study design, data collection and analysis, decision to publish, or preparation of the manuscript."

If this statement is not correct you must amend it as needed.

Please include this amended Role of Funder statement in your cover letter; we will change the online submission form on your behalf.

*We have added the required information that the funders had no role in study design, data collection and analysis, decision to publish, or preparation of the manuscript. We have also added this to the cover letter.*

Reviewer #1: In the title the authors write "k-mode clustering" but in the method section it states "K-medoids" without mentioning "k-mode"(line 207). It should be noted that these terms and the corresponding algorithms are very different.

*We have modified the title accordingly*

title - What are these "groups of production parameters and bulk tank milk antibody status"? I don't see any groups of parameters reported. The groups should be of the farms according to the context.

*Yes, the groups are indeed the farms. in fact, groups of farms were modelled. We have modified the title, to further clarify.*

line 67, This sentence is confusing. what is the difference between the terms "cluster" and "group" in this sentence. For clustering analyses, the cluster is the group.

*We can understand the confusion in this regard and have modified the sentence.*

line 191, "Missing values were excluded from" -> Farms with missing values were excluded

*Effected.*

Line 192, "Apart from" could mean "except for" or " in addition to". Is antibody status in or not in the model? I think " in addition to" is better here.

*Thank you. We have modified the expression in alignment with your remark.*

line 213 "PAM replaces centroids with medoids"-> PAM replaces means with medoids

*Effected.*

line 218 "Classification of Clusters by means of Random Forest". Clustering analyses is for unsupervised classification, I don't see why clusters need to be classified again. Besides, there are only 2 cluster reported (figure 1), how could these be classified again?

*The cluster analysis solely grouped the farms together ion the first place. As you said, this is already a sort of classification by sorting similar data point (i.e. farms together). However, after this initial clustering classification (i.e. assignment of farms to one of the two clusters within each study region), we only know that within each study region apparently there are two different groups of farms represented by the clusters. So far, so good. Our intention was to further understand what was behind these two groups of farms meaning how can the farms be characterised or rather how can both clusters be characterised or differentiated based on further variables. Therefore, after the initial sorting of farms into the two clusters, we took external/further variables which initially were not used for cluster creation and which were potentially related to the input variables of the cluster analyses (please refer to the network structure where we provided illustration on this) in order to find out how these external/further factors could help characterising the clusters. This was done using the random forest approach which allowed for the identification of factors that could characterise and differentiate the initially formed clusters and for finding the most important variables reflecting differences among clusters. To avoid confusion, we have replaced the term “classification” with the term “characterisation" in order to render it more clearly. Furthermore, we have added some more information to the first sentence of this paragraph.*

Reviewer #2: The manuscript describes a strong and novel work that used k-mode clustering to separate the internal features of the different diary farms and used random forest to analyze features that are important to the cluster separation. The authors used very rigorous statistical approaches in data acquisition, cleaning, aggregation, and analysis. The authors clearly demonstrated the methodology and results, and come to a well-supported conclusion that the antibody status of two major internal parasites in diary cows strongly affects the milk yield and nutrition content. The work thoroughly covered a vast number of farms over major diary production regions in Germany. The conclusion was also supported by relevant studies and field. In addition to the scientific insight that brings direct economical benefits, the manuscript demonstrated the feasibility of using un-supervised methods to perform non-biased analyze on the topic. Therefore, I strongly recommend publication of the work.

*We kindly appreciate this very positive and supportive feedback and are happy about the support.*
