# Supplementary material for: A machine learning approach using partitioning around medoids clustering and random forest classification to model groups of farms in regard to production parameters and bulk tank milk antibody status of two major internal parasites in dairy cows
Source: PLoS One. 2022 Jul 11;17(7):e0271413. doi: 10.1371/journal.pone.0271413 (PMC9273072; doi:10.1371/journal.pone.0271413)
Supplement: Attachment — Submitted filename: Response to Reviewers.docx [file pone.0271413.s013.docx]

Reviewer #2: The manuscript is strong. The work applied an unsupervised approach that naturally separated the studied farms by positive/negative of common bacterial antibodies. With further feature analysis, the work identified the variables that are associated with infections as well as milk yield. The work is of important direct economical impact and is based on sound statistics. Therefore, I recommend the publication of the work.

*Dear Reviewer 2, we again kindly appreciate your support and positive appraisal of this article.*

Reviewer #3: The results presented in this paper are interesting and of practical importance in identifying biologically relevant differences between farms positive for F. hepatica or O. ostertagi, respectively and negative farms using farm-level bulk tank milk. This important in the veterinary field.

*Dear Reviewer 3, thank you very much for the positive and constructive feedback to our work. We have the impression that even though they are minor, the points you have raised are important to be addressed and will considerably improve and round up this article.*

The revisions suggested are minor and involve some additional explanations, plots, and/or statistical procedures.

Minor Revisions and comments:

1. p. 9, line 200: The authors have chosen the Gower’s distance matrix for clustering stating that it is the most common distance matrix for a mix of categorical and continuous values and cite 2 papers (28, 29). It is probably the first distance measure proposed in 1971, but there are currently many more choices. An unsupervised random forest can be used to obtain a proximity matrix that could also be used for clustering. A random forest easily handles mixed types of data. See Conrad and Bailey 2015 PLoS One paper on clustering Cystic Fibrosis patients, for an example. It will probability not make much difference in the clustering results, but the authors should be aware of other measures.

*Thank you. We have changed the wording of this sentence*

2. p. 15, line 303: For all cluster analyses, the silhouette method selected 2 clusters to be optimal …

Authors should provide an average silhouette plot that shows that 2 clusters are optimal. This is because in the Cluster Analyses Section and the description of Fig 1 (F. hepatica cluster analyses) and Fig 2 (O. ostertagi cluster analyses) there were often a “majority cluster” and a “mixed cluster”. It would be very interesting to know that if k=3 clusters were chosen (assuming that the average silhouette plot showed that 2 or 3 clusters were reasonable choices), if the mixed cluster divided into 2 additional more homogeneous or identifiable clusters.

*We have provided all silhouette plots for every single one of the cluster analyses. We agree with you that it would have been very interesting -given the constant presence of a «majority cluster» and a «mixed cluster» - to know that if k=3 clusters were chosen (assuming that the average silhouette plot showed that 2 or 3 clusters were reasonable choices), if the mixed cluster divided into 2 additional more homogeneous or identifiable clusters. The silhouette plots yet very unequivocally demonstrated that for the underlying data set a 2-cluster solution appeared to be statistically most appropriate, as the objects match best with their own cluster and poorly with other clusters indicated by a silhouette score of > 0.6. In opposite to that, the silhouette score for a k=3 cluster solution already visibly appeared to be considerably lower. Considering then the subsequent descriptive results from the clustering as well as the random forest results and including biological reasoning in the consideration, the selection of two clusters seems even more plausible and strengthens the work in itself. By providing this piece of information and including all silhouette plots as additional files to this manuscript, we believe that the plausibility, transparency, and traceability of our analyses is ensured.*

3. p. 27-28 Section: Classification of Clusters by means of Random Forest
Results of a supervised random forest and variable importance plots are given in
Fig 3 (F. hepatica for North (Fig 3 A) and South (Fig 3 B)) and Fig 4 (O. ostertagi for North (Fig 4 A) and East (Fig 4 B) and South (Fig 4 C)).

Statements are made about what variable are “most important”. This is obtained from the ranking of the variables. There is an rfPermute package that will perform a permutation test and provide estimated permutation p-values for the importance metric of the random forest by permuting the response variable. This would allow the authors to make statements about important variables with a reasonable p-value cut-off. This will strengthen the statements about identifying variables that are most important in classifying or separating the clusters.

*We appreciate this very useful suggestion. We have now included a permutation test as suggested using the rfPermute package (also updated in the supplementary file of R packages used). Based on the results, we have made modifications in the results and discussion section (and a short remark in the Materials & Methods Section) in order to present all relevant variables discriminating clusters based on these p values. We are convinced that this course of action has had a very positive impact on the quality of this work and has increased the strength of the conclusions drawn from our analyses. Therefore, we kindly appreciate your comment inciting us to look into this direction.*

4. p. 2: In the Abstract it is stated
“Across all study regions, co-infections with F. hepatica or O. ostertagi, respectively, farming type, and pasture access appeared to be the most important factors discriminating clusters (i.e.39 farms). Furthermore, herd size, BCS, and stage of lactation were relevant criteria distinguishing clusters.” Permutation p-values will allow the statement to be made based using a p-value cut-off.

*We have now included the estimation of permutation p values within the manuscript to support this statement based on p values. In order to comply with the journal`s guidelines specifically concerning abstract length, which should not exceed 300 words, we have refrained from including these p values into the abstract.*

5. Discussion:
p. 29, line 477: Authors state “Moreover, the current work is probably the first of its kind to implement a k-medoids cluster algorithm and PAM.” Please see and cite:

DJ Conrad, BA Bailey, 2015
Multidimensional clinical phenotyping of an adult cystic fibrosis patient population
PLoS One 10 (3), e0122705

DJ Conrad, J Billings, C Teneback, J Koff, D Rosenbluth, BA Bailey, ..., 2021
Multi-dimensional clinical phenotyping of a national cohort of adult cystic fibrosis patients
Journal of Cystic Fibrosis 20 (1), 91-96

*We have rephrased the sentence and included both references*
